# Catalytic Activity of Oxidized Carbon Black and Graphene Oxide for the Crosslinking of Epoxy Resins

**DOI:** 10.3390/polym9040133

**Published:** 2017-04-07

**Authors:** Maria Rosaria Acocella, Carola Esposito Corcione, Antonella Giuri, Mario Maggio, Gaetano Guerra, Alfonso Maffezzoli

**Affiliations:** 1 Dipartimento di Chimica e Biologia e Unità di Ricerca INSTM, Università di Salerno, 84084 Fisciano (SA), Italy; mariomaggiochem@gmail.com (M.M.); gguerra@unisa.it (G.G.); 2 Dipartimento di Ingegneria dell’Innovazione, Università del Salento, 73100 Lecce, Italy; antonella.giuri@unisalento.it (A.G.); alfonso.maffezzoli@unisalento.it (A.M.)

**Keywords:** carbon black oxidation, Differential Scanning Calorimetry, gel time

## Abstract

This article compares the catalytic activities of oxidized carbon black (oCB) and graphene oxide (eGO) samples on the kinetics of a reaction of diglycidyl ether of bisphenol A (DGEBA) with a diamine, leading to crosslinked insoluble networks. The study is mainly conducted by rheometry and Differential Scanning Calorimetry (DSC). Following the same oxidation procedure, CB samples are more efficiently oxidized than graphite samples. For instance, CB and graphite samples with high specific surface areas (151 and 308 m^2^/g), as oxidized by the Hummers’ method, exhibit O/C wt/wt ratios of 0.91 and 0.62, respectively. Due to the higher oxidation levels, these oCB samples exhibit a higher catalytic activity toward the curing of epoxy resins than fully exfoliated graphene oxide.

## 1. Introduction

Many recent studies have shown the catalytic activity of graphene oxide (eGO) [1,2,3,4,5,6,7,8,9,10,11,12,13,14,15] and graphene (G) [16,17,18] toward numerous organic reactions. For several organic reactions, like the Mukayiama-Michael [7] or Friedel-Crafts reaction [18], similar catalytic activities have been observed for GO and G. In particular, for the Mukayiama-Michael reaction, it has been clearly established by DFT calculations that the catalytic activity is mainly due to supramolecular π-stacking interactions operating between the catalyst and the substrates [7]. For most organic reactions, like the Mukayiama-Aldol reaction [8] or esterification reactions [19], the catalytic activities of GO are definitely higher than for G (often negligible). In these cases, of course, the GO catalytic activity has been attributed to its functional groups, mainly those which are acidic.

The GO catalysis of ring-opening reactions in the amine crosslinking of epoxy resins [10,20,21] is included in this second class. In fact, high specific surface area graphite, as well as highly reduced graphene oxide, present negligible catalytic activity.

Very recently, direct kinetic studies, including a full chemical characterization and quantitative evaluation of the low molecular mass products for reactions of diglycidyl ether of bisphenol A (DGEBA) with primary and secondary monoamines and alcohols, have shown that GO and exfoliated graphite oxide (eGO) are able to catalyze primary amine-epoxy, secondary amine-epoxy, and hydroxyl-epoxy additions [20]. In the same paper, it has been shown that non-oxidized graphene surfaces are only able to catalyze the first two reactions and not hydroxyl-epoxy additions, which are also needed for the completion of the crosslinking reaction [20].

For many decades, it has also been known that oxidized carbon black (oCB) is able to affect the cure behavior of epoxy resin systems [22,23]. In a recent paper, the WAXD patterns of oCB samples were interpreted by a disordered spatial arrangement of graphene oxide layers, exhibiting short in-plane correlation lengths [24].

In the present paper, the catalytic activity of eGO and oCB samples, exhibiting very different oxygen contents, in the amine crosslinking of DGEBA epoxy resins, the most industrially relevant epoxy resin, is compared. The possible catalytic behavior has been mainly explored by indirect phenomenological approaches [25], based on differential scanning calorimetry (DSC) and the rheological evaluation of viscosity increases.

## 2. Materials and Methods

### 2.1. Materials

**Scheme 1:**
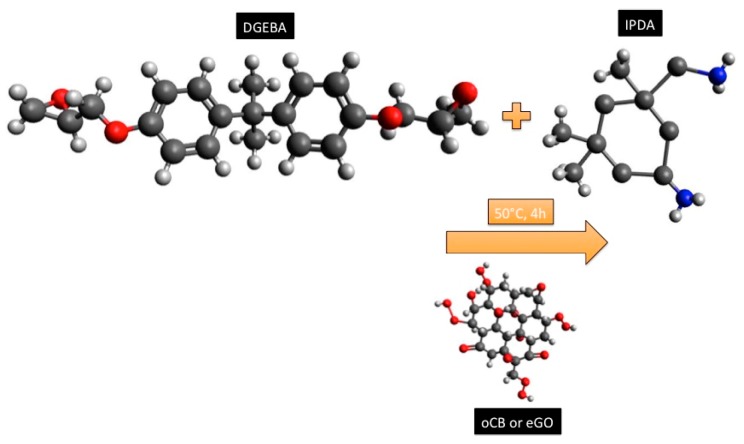
Macromolecular structure of the reactants.

Diglycidyl ether of bisphenol A (DGEBA), supplied by Resolution Performance Products (Houston, TX, USA) with the commercial name Epikote 828, is characterized by an epoxy equivalent weight (EEW) of 190 g/eq., corresponding to an average molecular weight (MwEpikote828) of 380 g/mol. Since neat DGEBA is characterized by an EEW = 170 g/eq., which corresponds to an average molecular weight (MwDGEBA) of 340 g/mol, as shown in Scheme 1, Epikote 828 is a mixture of neat DGEBA and its oligomers.

The curing agent, purchased by Sigma Aldrich (Milan, Italy), is isophorondiamine (IPDA), used without any further purification. IPDA is characterized by a Hydrogen Equivalent Weight, HEW = 43. It was selected since it is a typical hardener used for the fabrication of epoxy-based composites. According to the stoichiometric ratio, 22 parts of hardener (i.e., 22 phr) were added to 100 parts of DGEBA. The molecular structure of the components of the thermoset resin, as well as the crosslinking conditions, are shown in Scheme 1.

Synthetic Graphite 8427^®^ with a high specific surface area (HSAG, of about 308 m^2^/g) and a high shape anisotropy of the crystallites [26], was purchased from Asbury Graphite Mills Inc. (Asbury, NJ, USA).

The used carbon black samples, with specific surface areas of 36, 125, and 151 m^2^/g, respectively, were purchased from Cabot (Boston, MA, USA).

### 2.2. Preparation Procedures

#### 2.2.1. Preparation of Oxidized Graphitic Materials

Graphite Oxide (GO) and oxidized Carbon Black (oCB), were prepared by the Hummers’ method of high specific surface area graphite and commercial carbon black samples [27]. A total of 120 mL of sulfuric acid and 2.5 g of sodium nitrate were introduced into a 2000 mL three-neck round bottomed flask immersed into an ice bath and 5 g of carbon was added, with magnetic stirring. After obtaining a uniform dispersion, 15 g of potassium permanganate was added to the sample very slowly, to minimize the risk of explosion. The reaction mixture was thus heated to 35 °C and stirred for 24 h. Deionized water (700 mL) was added to the resulting black and dark green slurry in small amounts, for carbon black and graphite, respectively, under stirring and, finally, 5 mL of H_2_O_2_ (30 wt %) was gradually added. The obtained sample was poured into 7 L of deionized water, and then centrifuged at 10,000 rpm for 15 min with a Awel MF-48R centrifuge (Nuaire Plymouth, MN, USA). The isolated GO and oCB powders were washed twice with 100 mL of a 5 wt % HCl aqueous solution, and subsequently washed with 500 mL of deionized water. Finally, powders were dried at 60 °C for 12 h. About 6 g of all oCB samples and 7.5 g of GO powder were obtained. The obtained Oxygen/Carbon weight ratio was 0.62 for graphite oxide, and 0.16, 0.83, and 0.91 for oCB as obtained from CB samples exhibiting largely different BET specific surface areas (in the range 33–151 m^2^/g) [24].

Exfoliated graphite oxide (eGO) samples were prepared in a planetary ball mill (Fritsch Pulverisette 7, (FRITSCH GmbH, Idar-Oberstein, Germany) for 2 h, with a milling speed of 500 rpm, using 125 mL of GO powder which was added to a ceramic jar (inner diameter of 75 mm) containing stainless steel balls (10 mm in diameter), with a ball-to-powder mass ratio of 10:1.

#### 2.2.2. Dispersion of Graphite-Based Nanofillers and the Cure of the Epoxy Resin

The dispersion of the graphite-based fillers in the epoxy matrix was performed by adopting the solvent swelling method, previously reported in [28]. Each filler was dispersed in the polymer, according to the following steps. After the dispersion of each filler in acetone, the filler-solvent mixture was added to the epoxy resin and stirred for 5 h at 80 °C and 400 rpm, until the complete evaporation of the solvent. The filler-epoxy mixture was subsequently degassed under vacuum at *T* = 60 °C. A total of 22 phr of IPDA were finally added to the filler/resin mixture. A filler amount equal to 3 wt % was always added to the epoxy matrix. 

### 2.3. Characterization Techniques

#### 2.3.1. Elemental Analysis

Elemental analysis was performed with a Thermo FlashEA 1112 Series CHNS-O analyzer (Thermo Ficher Scientific, Waltham, MA, USA), after pretreating the samples in an oven at 100 °C for 12 h.

#### 2.3.2. Wide-Angle X-ray Diffraction

Wide-angle X-ray diffraction (WAXD) patterns were obtained by an automatic Bruker D8 Advance diffractometer (Bruker, MA, USA), in reflection, at 35 KV and 40 mA, using nickel filtered Cu-kα radiation (1.5418 Å). The *d*-spacings were calculated using Bragg’s law and the observed integral breadths (βobs) were determined by a fit with a Lorentzian function of the intensity corrected diffraction patterns. The instrumental broadening (βinst) was also determined by the fitting of the Lorentzian function to line profiles of a standard silicon powder 325 mesh (99%)*.* For each observed reflection, the corrected integral breadths were determined by subtracting the instrumental broadening of the closest silicon reflection from the observed integral breadths, β = βobs − βinst. The correlation lengths (*D*) were determined using Scherrer’s equation.

(1) $$D=\frac{K\lambda }{\beta \cos\theta }$$

where λ is the wavelength of the incident X-rays and θ is the diffraction angle, assuming the Scherrer constant *K* = 1.

#### 2.3.3. Thermogravimetric Analysis

The thermogravimetric (TG) analysis was carried out on a TG 209 F1, manufactured by TA Instruments (Waters, MA, USA), from 20 to 800 °C at a heating rate of 10 °C/min, under 20 mL/min N_2_ flow. 

#### 2.3.4. Infrared Spectroscopy

The FTIR spectra were obtained at a resolution of 2.0 cm^−1^ with a FTIR -BRUKER Vertex70, (Bruker, MA, USA) spectrometer equipped with a deuterated triglycine sulfate (DTGS) detector and a KBr beam splitter, using KBr pellets prepared by mixing 1 mg of sample in 100 mg of KBr. The frequency scale was internally calibrated to 0.01 cm^−1^ using a He−Ne laser. A total of 32 scans were signal averaged to reduce the noise. The spectra acquisition was in the 4000–450 cm^−1^ wavenumber range.

#### 2.3.5. BET Specific Surface Areas

Specific surface areas of the carbon and oxidized carbon samples were measured by nitrogen adsorption at liquid nitrogen temperature (77 K) with a Nova Quantachrome 4200e instrument. Before the adsorption measurements, samples were degassed at 60 °C under vacuum for 24 h. The specific surface area values were determined by using 11-point BET analysis (Quantachrome Instruments, Boynton Beach, FL, USA).

#### 2.3.6. Rheometry

The rheological properties of the filled and unfilled mixtures were evaluated by monitoring the evolution of the storage modulus, **G**′, and the loss modulus, **G″**, as functions of time. Measurements were made using a strain-controlled rheometer (Ares, Rheometric Scientific, Piscataway, NJ, USA), adopting a parallel plate geometry characterized by plates with a radius of 12.5 mm. Three isothermal tests at 50 °C were performed on each sample, using a frequency of 1 Hz and a deformation of 10%. 

The dynamic-mechanical properties of a curing system depend on the degree of reaction, dramatically changing when the gel point is approached. The time to gelation or gel time (*t*_g_) was determined according to the literature, as the cross between the **G**′ and **G″** curves [29,30]. Each test was performed until gelation was observed.

Finally, it must be underlined that the gel time is observed for a constant value of the degree of the reaction, according to Flory [31], and hence it represents a fast way to compare the rate of the reaction of thermosetting resins with the same reagents and under different conditions, such as those considering temperature or catalysis.

#### 2.3.7. Differential Scanning Calorimetry

The curing reaction of filled and unfilled epoxy resin was measured using a differential scanning calorimeter (DSC) supplied by Mettler Toledo 622 (Columbus, OH, USA). Dynamic DSC scans were performed on liquid samples from 20 to 250 °C at 10 °C/min, under nitrogen atmosphere. Isothermal DSC scans were also performed at 50 °C for 4 h, under nitrogen atmosphere, performing at least three repetitions. The reaction was considered to be complete when the heat flow curve leveled off to a constant value. The area under the exothermal curve, based on an extrapolated horizontal baseline aligned to the asymptotic value of the DSC signal at the end of the reaction, was used to calculate the heat of the reaction, Δ*H* (J/g), at the test temperature. 

## 3. Results

### 3.1. Characterization of the Carbon Nanofillers

An elemental analysis of the carbon-based nanofillers used in the present study is reported in Table 1.

In agreement with a previous report [24], the O/C ratio of the considered oCB samples, for the same oxidation procedure, markedly increases (0.16, 0.83, and 0.91) with the specific surface area of the starting CB samples (36, 125, and 151 m^2^/g, respectively), with an average size of 50–100 nm. These three oCB samples thereafter will be named oCB-1, oCB-2, and oCB-3, respectively. For the same oxidation procedure, the eGO sample, i.e., the graphene oxide sample as obtained by the ball-milling of graphite oxide, exhibits a rather low O/C ratio (0.62), irrespective of the very high specific surface area of the starting graphite (308 m^2^/g).

**Figure 1:**
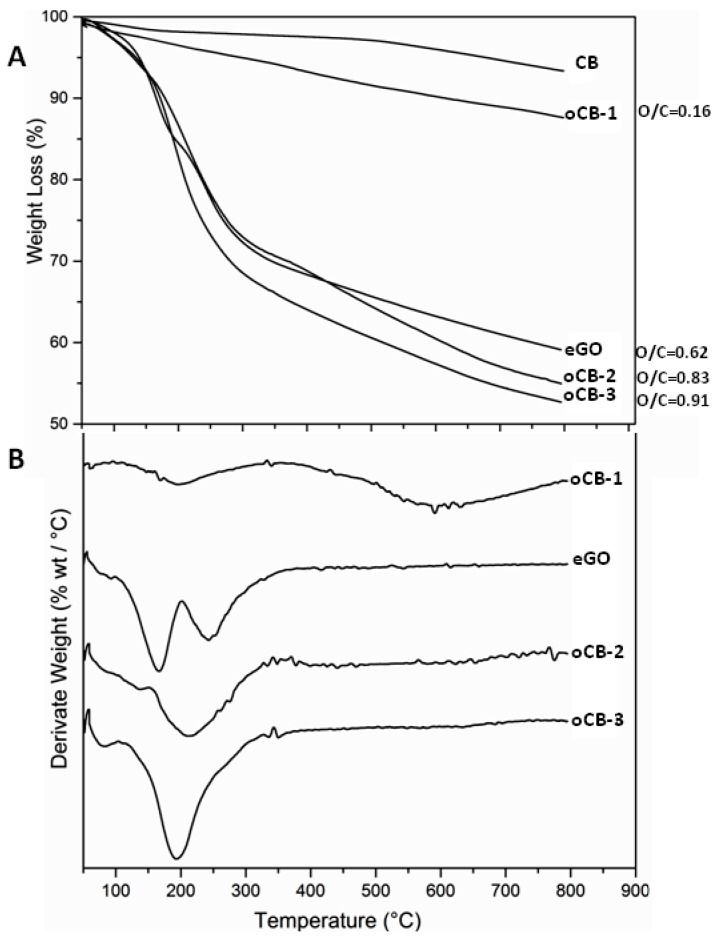
TGA scans at 10 °C/min after drying at 100 °C in the TGA apparatus, of starting Carbon black (CB) and all the considered carbon nanofillers: oCB-1; eGO; oCB-2; oCB-3. (**A**) TGA; (**B**) DTGA. Close to each curve, their O/C molar ratio is indicated.

The large difference in the degree of oxidation of the considered nanofillers is also clearly shown by the TGA scans of Figure 1. 

**Figure 2:**
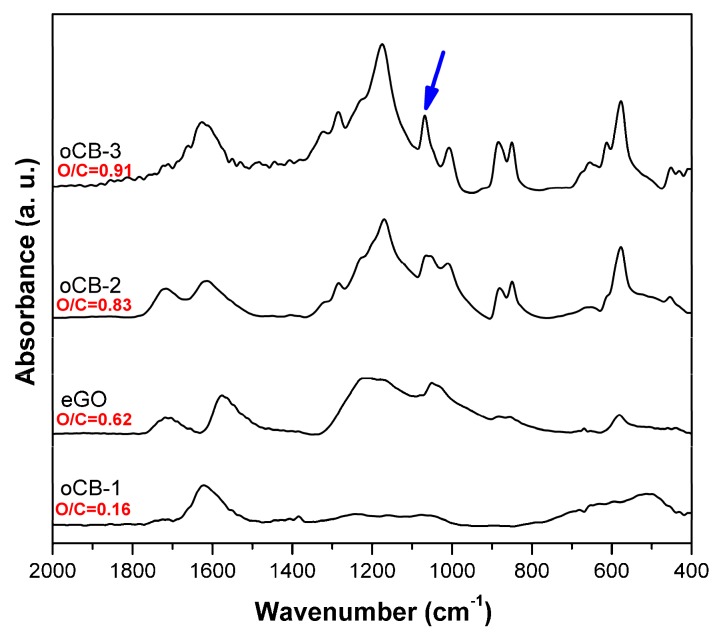
FTIR spectra in the range 2000–400 cm^−1^ of the considered carbon nanofillers: oCB-1; eGO; oCB-2; oCB-3. Close to each curve, their O/C molar ratio is indicated.

Information relative to the functional groups present in the different nanofillers is obtained from the FTIR spectra (Figure 2).

In particular, as the O/C ratio increases, many vibrational peaks appear in the 1300–900 cm^−1^ range (mainly referring to the C–O stretching of the ether, epoxy, alcoholic, and carboxylic groups), becoming progressively more defined. Moreover, the well-defined vibrational peak located at 1069 cm^−1^ (indicated with an arrow in Figure 2) reveals the contribution of an increased amount of the sulfonic group [32,33].

**Figure 3:**
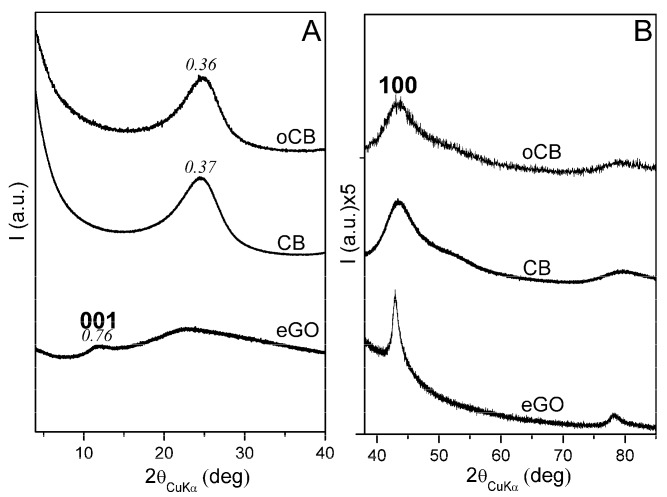
X-ray diffraction patterns (CuKα) of: graphene oxide (eGO, with O/C, wt/wt = 0.62); CB sample with the highest BET specific surface area; this CB after oxidation (oCB-3, O/C, wt/wt = 0.91). Patterns in the 2θ range 4–40° (**A**) and enlarged patterns in the 2θ range 40–85° (**B**).

Information on the structural organization of the oxidized samples has also been obtained by WAXD. Figure 3 compares the WAXD patterns of the graphene oxide sample and of the carbon black sample with the highest specific surface area and of the corresponding mostly oxidized sample (oCB-3). The WAXD pattern of graphene oxide (lower pattern in Figure 3) shows the maintenance of hk0 (110 and 100) graphite reflections and the presence of a broad halo centred at 2θ = 24°. This indicates the presence of an in-plane graphitic order and the nearly complete absence of a packing order between the graphene oxide layers.

The WAXD pattern of the CB sample with the highest BET specific surface area (intermediate pattern in Figure 3) is very similar to those of all the considered carbon black samples and poorly changes after extensive oxidation up to O/C = 0.91 (upper pattern in Figure 3) [24]. As expected, the in-plane graphitic order for the oCB samples is much lower than for the eGO sample. For instance, the in-plane correlation length (as evaluated from the broadness of the 100 reflection) decreases from 11.1–11.3 nm to 2.1–2.3 nm, passing from GO to oCB. The amorphous halo, on the contrary, is definitely broader for the GO sample rather than for the oCB samples, with the half-height width reducing from 10.1° to 4.5°. The occurrence of amorphous halos which are much broader for eGO than for CB and oCB, could be rationalized by a broader distribution of interatomic distances, which is possibly determined by the disordered spatial disposition of the larger layers.

### 3.2. Gel Time of the Epoxy Resin Crosslinking Reaction by the Rheometer

**Figure 4:**
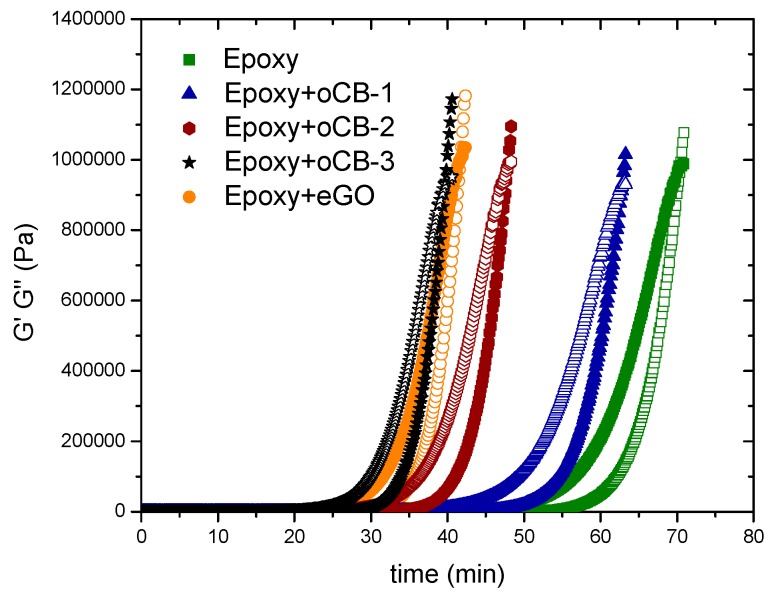
Storage modulus (**G**′, empty symbols) and loss modulus (**G**″, full symbols) versus time for the DGEBA-IPDA epoxy resin, pure (**green**) or with 3 wt % of different carbon nanofillers: oCB-1 (O/C, wt/wt = 0.16) (**blue**); oCB-2 (O/C, wt/wt = 0.83 (**wine**); oCB-3 (O/C, wt/wt = 0.91) (**black**); eGO (O/C, wt/wt = 0.62) (**orange**).

Carbon nanofillers thoroughly characterized in the previous section have been tested in model reactive epoxy resins, close to those used in practical applications, as possible catalysts for the crosslinking of DGEBA epoxy resins with amines. The cure reaction was studied by isothermal rheological measurements, at 50 °C. The storage modulus, **G**′ (empty symbols), and the loss modulus, **G″** (full symbols), of unfilled and filled compounds, as determined by forced harmonic oscillation measurements, are reported in Figure 4 as functions of time.

The gel time (*t*_gel_), measured as the cross between the **G**′ and **G**″ curves of the neat resin and of the nanocomposites, is reported in Table 2. 

The gel time of the neat epoxy resin (*t*_g_ = 70.5 min) is progressively reduced with the degree of oxidation of oCB and is nearly halved in the presence of the oCB sample with O/C=0.91 (*t*_g_ = 39.7 min). An effective reduction of the gel time is also observed with eGO (O/C = 0.62, *t*_g_ = 41.7 min). The gel time represents the time to reach a given value of the degree of reaction once the stoichiometry is defined. In this case, according to the Flory equation [31], the reaction of a bifunctional epoxy monomer with a tetrafunctional amine (neglecting side reactions with OH groups) leads to a degree of reaction at gelation of 0.58. Since the lower the gel time, the higher the reaction rate, these data clearly confirm a catalytic activity of the oxidized groups on the graphitic layers on the epoxy crosslinking. 

### 3.3. DSC Heating Scans of Unfilled and Filled Epoxy Resins

**Figure 5:**
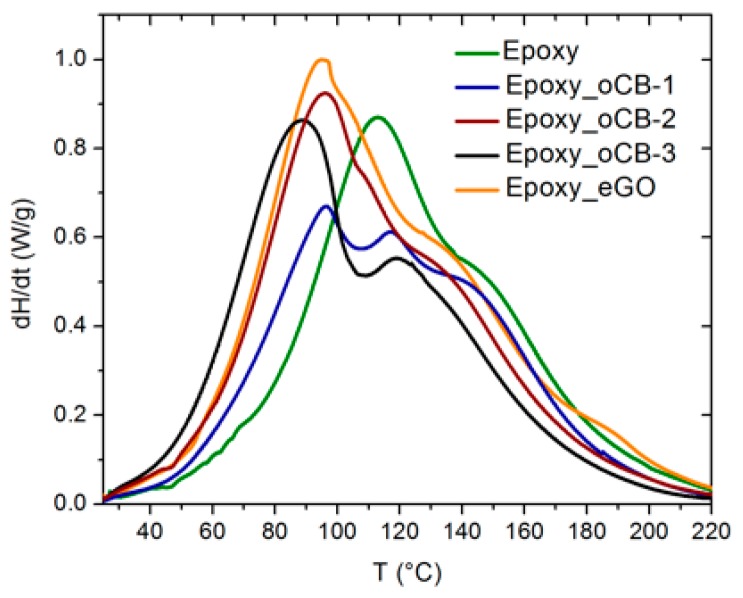
DSC scans (heating rate = 10 °C/min) for the pure epoxy resin (**green**) and for composite resins with 3 wt % of: oCB-1 (**blue**); oCB-2 (**wine**); oCB-3 (**black**); eGO (**orange**).

DSC scans, performed on the neat epoxy resin and on resins filled with 3 wt % of the considered carbon nanofillers, at a heating rate of 10 °C/min, are compared in Figure 5. 

The scan of the neat resin (green curve in Figure 5) presents, beside the principal exothermic peak at 114 °C, a shoulder (roughly located at 145 °C) that could be possibly due to a hydroxyl-epoxy addition (etherification) reaction [20]. For the compound with the poorly oxidized CB sample (oCB-1), two peaks are clearly distinguished, with a more intense one at 96 °C, which clearly indicates the occurrence of some catalysis toward the etherification reaction [10,20]. In the absence of any catalyst, the amine addition is strongly favored with respect to etherification. However, oxidized graphites are catalysts of both reactions, as formerly demonstrated [10,20], acting first on primary and secondary amine additions, and then on etherification. This last reaction occurs when a gelled network is formed and hence it is also affected by the mobility of chain segments, which depends on the degree of crosslinking. Therefore, when more amine hydrogens react (first peak in Figure 5), more hydroxyl are produced, but also more steric hindrance is expected at higher temperatures, when etherification is favored. Therefore, the intensity of the first peak progressively increases at the expense of the higher temperature peaks as the oxidation degree of the carbon nanofillers increases. Moreover, for the mostly oxidized sample (oCB-3), this peak corresponding to the amine catalyzed reaction is also centered at the lowest temperature (≈ 89 °C). The fraction of the heat of the reaction associated with the amine catalyzed reaction, assessed by peak deconvolution for the DSC scans of Figure 5, is shown in Table 3. The observed shift to lower temperatures of the first exothermic peak indicates a relevant catalytic activity of graphite oxide toward the amine-epoxy reaction. 

For the exothermic phenomena of Figure 5, the total heat of the crosslinking reaction per mass of resin (∆*H*_U_) and the position of the main peak (*T*_peak_) are also listed in Table 3. 

It is apparent that the *T*_peak_, as well as the heat of the reaction, increases with the degree of oxidation of the carbon nanofillers. The number of reacte epoxy groups, indicated by ∆*H*_U_ in Table 3, also increases with the degree of oxidation of nanographite. 

### 3.4. Kinetics of the Epoxy Resin Crosslinking Reaction by Isothermal DSC

**Figure 6:**
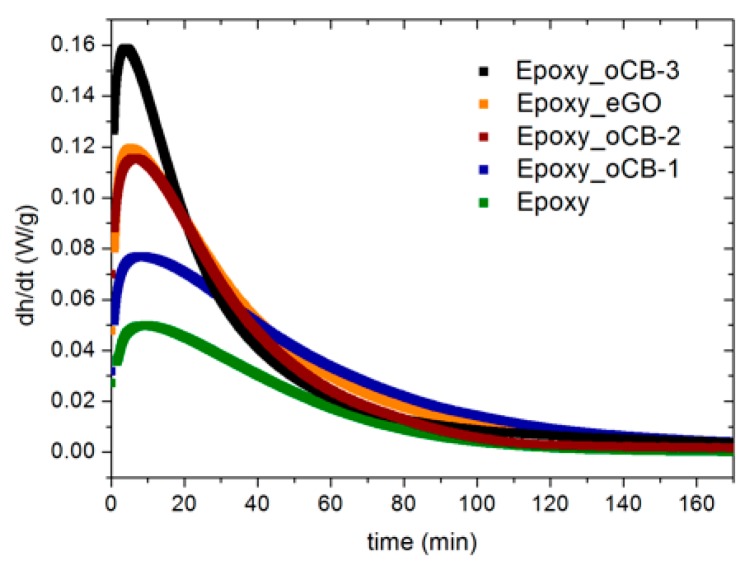
Isothermal DSC scans at 50 °C of the DGEBA-IPDA epoxy resin. The lowest curve (**green**) corresponds to the neat epoxy resin. The other curves correspond to the epoxy resin filled by 3 wt % of different nanofillers: oCB-1 (O/C, wt/wt = 0.16) (**blue**); oCB-2 (O/C, wt/wt = 0.83) (**wine**) oCB-3 (O/C, wt/wt = 0.91) (**black**); eGO (O/C, wt/wt = 0.62) (**orange**).

Isothermal DSC scans at 50 °C, for the considered epoxy resin, without fillers and with 3 wt % of different graphite-based nanofillers, are reported in Figure 6. A short time interval, usually about 30 s, is needed for temperature stabilization after the ramp at 40 °C/min used to reach the isothermal test temperature. The transition between dynamic and isothermal conditions is responsible for a jump in the heat flow, which means that the data obtained in the first 30 s of the DSC test are not reliable. For this reason, all isothermal curves were corrected, neglecting the data point in the first 30 s after the curing temperature is reached, and determining by linear extrapolation of the data points recorded between 30 and 60 s, the value of the heat flow at time = 0. These corrected isothermal DSC scans are shown in Figure 6 and the isothermal heat of the reaction and the time to reach the peak, calculated according to the procedure described in the Experimental section, are listed in Table 4, together with the residual heat of the reaction (∆*H*_residual_) and the glass transition temperature (*T*_g_) measured from a dynamic scan performed after the isothermal scan. 

As already observed for the gel time data of Table 3, the peak time (*t*_peak_) and the residual heat of the reaction of Table 4 are significantly reduced in the presence of eGO, oCB-1, and oCB-3. This further indicates the catalytic activity of oxidized graphitic layers on epoxide-amine reactions. 

Compounds with graphite-based nanofillers are also characterized by a significantly higher heat of reaction: neat epoxy resin is characterized by a reaction enthalpy of 158 J/g, increasing to 306 J/g when oCB-1 is added and 337 J/g in the presence of eGO and oCB-3. An increase of about 93% is already observed with oCB-1, i.e., the less oxidized CB. Because the overall crosslinking enthalpy for the unfilled and filled epoxy resins, as determined by DSC heating scans, are all in the range 310–380 J/g (see, next section), the low reaction enthalpy of the neat epoxy resin clearly indicates that in the absence of the carbon-based fillers, less than 50% of the crosslinking reaction occurs, even after long term treatments at 50 °C. Isothermal curing at 50 °C leads to vitrification, which is responsible for the end of the reaction, which depends on free volume relaxation [34,35,36,37,38], delayed with respect to chemical conversion when faster reactions occur, i.e., when graphite oxide is present. The graphite-based fillers promote an increase of the *T*_g_ of the neat resin from 54.7 to 64.6 °C for Epoxy + oCB-3; this last behavior influences the kinetics reaction, as confirmed by the decreasing of the residual heat of the reaction from 74.6 (neat resin) to 13.4 J/g (Epoxy + oCB-3). It is important to note that the sum of ∆*H* (measured from the isothermal scan) and ∆*H*_residual_ (measured from the subsequent dynamic scan) is comparable to the value of ∆*H*_U_ (measured from the dynamic scan) reported in Table 3.

**Figure 7:**
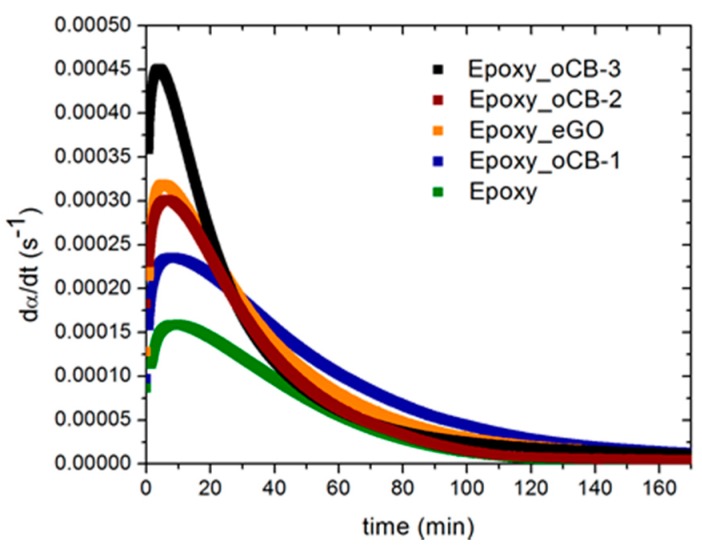
Comparison between *d*α/*d*t experimental curves of each system: epoxy resin filled by 3 wt % of different nanofillers: oCB-1 (O/C, wt/wt = 0.16) (**blue**); oCB-2 (O/C, wt/wt = 0.83) (**wine**); oCB-3 (O/C, wt/wt = 0.91) (**black**); eGO (O/C, wt/wt = 0.62) (**orange**).

In order to obtain further insight into the reaction mechanism, the rate of reaction, *d*α/*d*t, was calculated from the DSC scans, as:

(2) $$\frac{d\alpha }{dt}=\frac{1}{H_{U}}\left(\frac{dH}{dt}-BL\right)$$

where *BL* is the baseline, set as described above; *dH*/*dt* is the heat flow for unit mass shown in Figure 7; ∆*H*_U_ is the total heat of the reaction obtained by the integration of each peak of the dynamic scan reported in Figure 5, adopting the same baseline [39]; α represents the actual degree of reaction; and *d*α/*dt* is the rate of the reaction. α represents a phenomenological measure of the advancement of the curing reaction and is obtained by the integration of Equation (1). A comparison between the *d*α/*d*t experimental curves obtained by applying Equation (1) to the DSC scans of each system, is reported in Figure 7. 

Multiple events may occur simultaneously and lead to a very complicated reaction, and consequently, the use of multiple rate constants can provide more accurate modeling results. Kamal’s model [40] involves two rate constants and has been successfully applied to model a variety of resins:

(3) $$\frac{d\alpha }{dt}=(k_{1}+k_{2}\alpha^{m}){\left(1-\alpha \right)}^{n}$$

where *m* and *n* are the fitting parameters which do not depend on temperature, and *k*_1_ and *k*_2_ are temperature-dependent rate constants. The validity of Equation (3) is limited to the reactions for which the kinetics of bond formation is the only rate-controlling step in the curing process. While this is usually true in the early stage, species diffusion can become very slow and govern the curing reaction rate near and above the glass transition. To take into account the different cure rate controlling mechanisms and achieve a greater accuracy at high conversions, some modifications of the available cure kinetics models have been introduced by Cole [41,42], by adding a term to explicitly account for the shift from kinetics to diffusion control in an autocatalytic thermosetting resin system. The modified expression has the following form:

(4) $$\frac{d\alpha }{dt}=\frac{k\alpha^{m}{\left(1-\alpha \right)}^{n}}{1+e^{c\{\alpha -\left(\alpha_{c0}+\alpha_{ct}T\right)\}}}$$

where *c* is the diffusion constant and α*_c_*_0_ is the critical degree of cure at a temperature of absolute zero. The constant α*_ct_* accounts for an increase in the critical resin degree of cure with temperature. *K*, *m*, and *n* follow the same definitions as in previous equations. Both Cole and Kenny et al. [40,41,42], proposed a simpler way to account for vitrification as the cause of the end of a reaction at low curing temperatures [43,44]:

(5) $$\frac{d\alpha }{dt}=(k_{1}+k_{2}\alpha^{m}){\left(\alpha_{\max}-\alpha \right)}^{n}$$

where α_max_ is the maximum degree of cure at a given temperature due to the vitrification phenomenon observed in isothermal cure. The constants m and n are the reaction orders which do not depend on temperature, to be experimentally determined, and *k*_1_ and *k*_2_ are temperature-dependent rate constants. Equation (4) incorporates the term α_max_, so that the fractional conversion will not exceed the degree of cure associated with vitrification at a specific temperature. The isothermal rate of reaction data, reported in Figure 7, were interpolated with Equation (4), calculating α_max_, from:

(6) $$\alpha_{\max}=\frac{\Delta H_{\max}}{\Delta H_{U}}$$

where Δ*H*_max_ is the maximum heat of the reaction calculated from the isothermal scan, and Δ*H*_U_ is the ultimate heat released during a complete reaction, measured from the dynamic scan.

**Figure 8:**
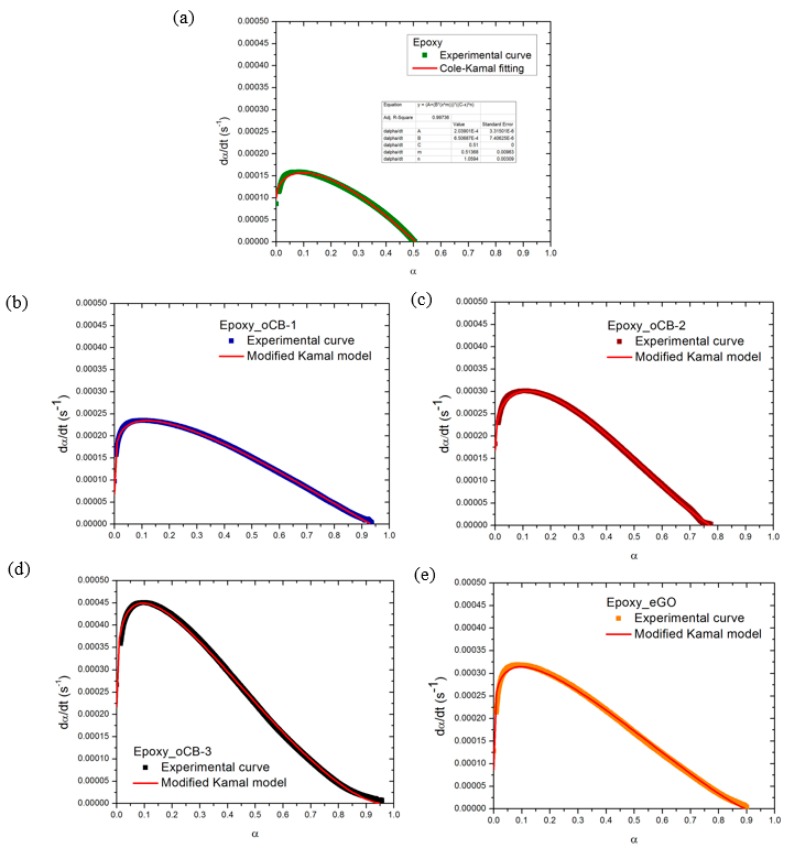
Comparison between Equation (6) model predictions and the experimental data of epoxy resins (**a**) filled by 3 wt % of different nanofillers; (**b**) oCB-1 (O/C, wt/wt = 0.16); (**c**) oCB-2 (O/C, wt/wt = 0.83); (**d**) oCB-3 (O/C, wt/wt = 0.91); (**e**) eGO (O/C, wt/wt = 0.62).

The constant *k*_1_ represents the initial rate of the reaction, i.e., *k*_1_ = *dα*/*dt* at *t* = 0. *k*_1_, readily obtained from experimental DSC data, and is reported for each sample in Table 5. A comparison between the kinetic model predictions and the experimental data for unfilled and filled systems, reported in Figure 8, shows a good agreement. The peak value, (*d*α/*dt*)_peak_, is reported for each experimental system in Table 5. 

The parameter *m* in Equation (5) is representative of the increase of the rate of the reaction at the beginning of the reaction (low values of α), before the peak. On the other hand, the parameter *n* in Equation (4) is dominant in the final part of the reaction (high values of α) [45] The autocatalytic effect, as measured by the low values of m, is higher than that of neat epoxy when oxidized graphites are used. 

The parameter n shown in Table 5 increases, changing from a value of about one to values higher than one when nanofillers are added to the resin, in particular, the highest value of *n* was found when oCB-3 is used. A possible explanation for this kinetic change can be addressed by looking at the differences in the peak time and the heat of the reaction. When the rate of the reaction is very fast, vitrification, responsible for the end of the reaction at this temperature, is delayed as a consequence of non-equilibrium conditions, corresponding to a difference between the actual and the equilibrium free volume, as occurs in a crosslinked system undergoing fast reactions [34,35,36,37,38]. At a high enough rate of reaction, the actual specific volume is not capable of following the equilibrium specific volume, corresponding to the instantaneous molecular configuration which depends on the amount of reacted groups. This effect explains why, in a faster reaction, a higher heat of reaction, i.e., a larger amount of reacted groups, is observed as a consequence of a delay in vitrification (see also ∆*H* in Table 4). Furthermore, at a higher rate of reaction, the specific volume is farther from its equilibrium value, and, close to the end of the reaction, this leads to a more rapid decrease in the rate of the reaction as a consequence of the vitrification driven reaction end point. The rate of the reaction decreases faster when *n* is higher: this is also evident from the last portion of the rate of reaction curves shown in Figure 7 (time > 30 min).

## 4. Conclusions

The influence of different graphite-based nanofillers (exfoliated graphite oxide and oxidized carbon black) on the epoxide ring opening reactions of diglycidyl ether of bisphenol A (DGEBA), the most used component of commercial epoxy resins, as induced by amines, has been studied.

For the same oxidation procedure, CB samples are more efficiently oxidized than graphite samples. For instance, CB and graphite samples with a high specific surface area (151 and 308 m^2^/g), as oxidized by the Hummers’ method, exhibit O/C wt/wt ratios equal to 0.91 and 0.62, respectively.

Rheological data at 50 °C confirm the occurrence of a catalytic behavior. In fact, the gel time is reduced by 41% and 44% in the presence of 3 wt % graphene oxide (eGO) and highly oxidized CB (oCB-3), respectively.

Isothermal DSC studies at 50 °C show that, correspondingly, the time for the exothermic peak is reduced for eGO and oCB-3 by 40% and 51%, respectively. The application of an auto-catalytic model to the experimental DSC data of each unfilled and filled system, to both neat epoxy resin and graphite-based epoxy nano-composites, leads to kinetic parameters coherent with the envisaged catalyzed reactions.

Dynamic DSC scans also clearly show the the catalytic activity of both eGO and oCB-3, with a fraction of the amine-epoxy catalyzed reaction (based on heating scans at 10 K/min), is close to 29% and 56%, respectively. Moreover, the main exothermal peak shifts from 113 to 95 and 89 °C, for eGO and oCB-3, respectively.

Hence, the oCB samples obtained from high specific surface area CB, due to their higher oxidation levels using the same oxidation procedure, exhibit catalytic activity toward the curing of epoxy resins which is definitely higher than for fully exfoliated graphene oxide. 

The reported results clearly confirm that oxidized carbon nanomaterials in epoxy-based composites not only act as nanofillers which improve the physical properties, but also as carbocatalysts, suitable for reducing the crosslinking (cure) temperature and/or time of the thermoset matrix.

## Figures and Tables

**Table 1:** Elemental analysis of the carbon-based nanofillers used in the study ^a^.

Nanofiller	C (wt %)	H (wt %)	O (wt %)	S (wt %)	O/C
oCB-1	85.9	0.2	13.75	<0.2	0.16
oCB-2	50.3	2.3	41.7	5.4	0.83
oCB-3	40.8	1.73	37.42	9.9	0.91
eGO	59.4	0.6	37.1	2.6	0.62

^a^ Elemental composition of anhydrous samples: The error in C and O content is close to 1 wt %, while the error in H content is close to 0.1 wt %; water content (as evaluated by TGA, after equilibration at room temperature in air with a relative humidity of 45%) was in the range of ±2 wt %, for all the examined samples.

**Table 2:** Gel time (*t*_gel_), measured at the intersection of the **G**′ and **G**″ curves of the neat resin and of the nanocomposites.

Sample	*t*_gel_ [min]
Epoxy	70.5 ± 5.6
Epoxy oCB-1	62.9 ± 4.4
Epoxy oCB-2	47.9 ± 3.6
Epoxy oCB-3	39.7 ± 2.0
Epoxy_eGO	41.7 ± 2.1

**Table 3:** Results of DSC scans at 10 °C/min, for the neat epoxy resin and epoxy resins with 3 wt % of different carbon nanofillers: enthalpy changes, normalized with respect to the resin weight (∆*H*_U_), peak temperature (*T*_peak_), and fraction of catalyzed reaction.

Sample	∆*H*_U_ (J/g)	*T*_peak_ °C	Amine catalyzed fraction (%)
Epoxy	313.9 ± 1.1	113.7 ± 0.9	0
Epoxy + oCB-1 (O/C = 0.16)	327.5 ± 2.2	96.5 ± 0.8	12
Epoxy + oCB-2 (O/C = 0.83)	383.8 ± 1.1	96.2 ± 0.7	34
Epoxy + oCB-3 (O/C = 0.91)	352.5 ± 1.7	88.7 ± 1.1	56
Epoxy + eGO (O/C = 0.62)	374.4 ± 2.3	95.0 ± 0.7	29

**Table 4:** Enthalpy, normalized with respect to the resin weight (∆*H*) and peak time (*t*_peak_) obtained by isothermal DSC scans at 50 °C, for the neat epoxy resin and the epoxy resin with different graphite-based nanofillers.

Sample	∆*H* (J/g)	*t*_peak_ (s)	∆*H*_residual_ (J/g)	*T*_g_ (°C)
Epoxy	158.0 ± 2.0	508 ± 1	74.6 ± 1.1	54.7
Epoxy + oCB-1	306.0 ± 18.0	504 ± 19	18.1 ± 0.9	66.3
Epoxy + oCB-2	297.7 ± 5.7	393 ± 6	62.6 ± 1.3	59.4
Epoxy + oCB-3	337.0 ± 27.0	249 ± 63	13.4 ± 0.4	64.6
Epoxy + eGO	337.0 ± 2.0	306 ± 1	26.2 ± 0.8	63.2

**Table 5:** *dα*/*dt*_peak_ (s^−1^) and kinetic model parameters.

Sample	(*d*α/*dt*)_peak_ (s^−1^)	α_max_	*m*	*n*	*k*_1_ (s^−1^) × 10^6^	*k*_2_ (s^−1^) × 10^6^
Epoxy	1.57 × 10^−4^	0.51	0.51	1.06	204	650
Epoxy + oCB-1	2.33 × 10^−4^	0.94	0.21	1.07	740	340
Epoxy + OCB-2	3.02 × 10^−4^	0.78	0.39	1.22	230	630
Epoxy + oCB-3	4.49 × 10^−4^	0.96	0.30	1.58	230	670
Epoxy + eGO	3.18 × 10^−4^	0.90	0.20	1.22	990	490
